# Supplemented with Astragalus dregs improves growth performance, immunity, and antioxidant capacity in fattening pigs

**DOI:** 10.1017/jns.2024.95

**Published:** 2025-01-17

**Authors:** Qi Guo, Jiayi Wang, Xuhan Wang, Hongyi Zhang, Jiajiao Xing

**Affiliations:** School of Biology and Pharmaceutical Engineering, Jilin Agricultural Science and Technology University, Jilin, China

**Keywords:** *Astragalus*, Feed additive, Production performance, Traditional Chinese medicine dregs

## Abstract

This study aimed to investigate the impact of Astragalus dregs — the residue after the extraction of principal active components — on the growth performance, antioxidant capacity, and immune function of fattening pigs. Twenty-four 130 days of age fattening pigs were randomly assigned to the control group and experimental group (supplemented with 10% Astragalus dregs). The production performance was evaluated by average daily gain (ADG), average daily feed intake (ADFI) and diarrhoea rates. Additionally, nutrient digestibility, blood biochemical parameters, antioxidant capacity, and immune function factors were analysed. The group supplemented with Astragalus dregs showed a trend towards improved ADG and ADFI and reduced diarrhoea rates (*p* > 0.05). Moreover, it significantly enhanced the digestibility of dry matter, crude protein, and ether extract (*p* < 0.05). Blood analysis revealed that globulin and total protein were increased, and glucose, cholesterol and triglyceride levels were decreased (*p* < 0.05) in the group supplemented with Astragalus dregs. The antioxidant capacity was significantly promoted by elevated T-AOC, GSH-px, and SOD activities and reduced malondialdehyde levels (*p* < 0.05). The immune function factors demonstrated that IgA, IgG, IgM, and anti-inflammatory cytokines IL-10 and IL-22 were significantly increased, meanwhile pro-inflammatory cytokines IL-2 and IL-6 were decreased (*p* < 0.05).

These findings indicate that Astragalus dregs, as a dietary supplement, may enhance growth performance, antioxidant capacity and immune function in fattening pigs. It is suggested that future studies should explore the optimal supplementation ratio of Astragalus dregs in pig diets.

## Introduction

Astragalus membranaceus, a traditional Chinese medicinal plant, has been utilised for centuries for its tonic and therapeutic properties^(1)^. The root of this leguminous plant is particularly valued for its multi-effect pharmacological activities, including anti-inflammatory, antioxidant, and immune-modulating effects. Modern pharmaceutical research indicated a series of bioactive compounds within Astragalus membranaceus, such as Astragalus polysaccharides, saponins, flavonoids, amino acids, and trace elements. Among these active components, Astragalus polysaccharides are commonly used as feed additives in pig production to enhance pig immunity and reduce piglet diarrhoea^(2–4)^. Astragalus dregs is the residue after the extraction of principal components. For technological constraints and focus on single compound extraction, the concentration of active ingredients within the Astragalus dregs accounts for nearly 70% of Astragalus membranaceus^(5)^. It has demonstrated that abundant active ingredients and nutrient substance *are in remaining* Astragalus dregs^(6)^.

In animal husbandry, Astragalus dregs, currently utilised as a functional feed additive, which exert effect in promoting growth performance and the apparent digestibility, improving animal immunity and meat quality, enhancing antioxidant capacity^(7,8)^.

The chemical profile of Astragalus dregs comprises polysaccharides, flavonoids, saponins, crude fibre, crude fat, and various trace elements^(9)^. The extraction rate of polysaccharides from Astragalus dregs is 12.93%, with a purity level of 93.27%. Astragaloside, a key bioactive saponin, the concentration of astragaloside within the Astragalus dregs accounts for 72.08% of the Astragalus^(10)^. Astragalus dregs also contain flavonoids such as calycosin, formononetin, and other isoflavonoids^(11)^.

Astragalus dregs, the residue after the extraction of principal components, have emerged as a promising functional feed additive with many potential benefits in animal nutrition. These dregs are rich in bioactive components such as Astragalus polysaccharides, saponins, and flavonoids, which have been extensively studied for their pharmacological properties^(12,13)^. The incorporation of these bioactive constituents into animal diets has been shown to enhance production performance, bolster immune function, resist stress, and activate digestive enzyme activities^(14)^.

Fermentation of Astragalus dregs is an approach employed to increase the release of bioactive substances^(15–18)^. The fermentation process can lead to the liberation of more bioactive compounds from Astragalus dregs; however, the origin of these substances — whether from the dregs themselves or as secondary metabolites from the fermentation culture — remains to be elucidated.

In animal husbandry, the impact of Astragalus dregs supplementation on growth performance, immune function, and antioxidant capacity, as well as the underlying immunomodulatory mechanisms, particularly in fattening pigs, has been less explored. Compared to weaned piglets, fattening pigs have greater growth demands and more robust intestinal absorptive functions, which can reflect the efficacy of Astragalus dregs supplementation.

In summary, Astragalus dregs, as an environmentally friendly functional feed additive have the potential to improve animal production performance, immune function, and antioxidant capabilities. However, further research is needed to reveal specific effects and mechanisms of Astragalus dregs supplementation in fattening pigs. Understanding these mechanisms could pave the way for the better utilisation of Astragalus dregs in animal feed additive.

## Materials and methods

### Animal experiment design

All animal welfare procedures were conducted in accordance with the Animal Experimental Committee of the Jilin Agricultural Science and Technology University (LL5C202303003).

Astragalus dregs is provided by Gansu Hongxin Pharmaceutical Co., Ltd. Table 2 are the main components of Astragalus Dregs.

The stall was dry, ventilated and was cleaned in the morning and evening. The temperature of stall was 20–25°C. The fattening pigs were fed by the same breeder. Twenty-four 130-day-old “Durec × Long” fattening pigs in good health were randomly divided into two groups, control and experimental groups, with 12 fattening pigs in each group (three replicate each group), resulting in four fattening pigs per replicate. The control group was fed the basal diet, the dietary composition and nutrient level were as Table 1. The experimental group was fed basal diet supplemented with 10% Astragalus dregs. Nutrient content of control and experimental diet (10% Astragalus dregs) were shown in Supplementary Table 1. All the fattening pigs had ad libitum access to diet and drinking water, and other management was carried out in accordance with the growth requirement for fattening pigs. The feed intake gain and diarrhoea rate of pigs in 0–28 days experiment period were recorded daily.

**Table 1.** Dietary composition, and nutrient level of the basal diet

Item	Amount
Ingredient, %	
Corn	65.00
Soybean meal, fermented	20.00
Bran	8.00
Fish meal	2.00
Calcium hydrogen phosphate	0.60
Sodium chloride	0.70
Limestone	0.70
Soybean oil	1.50
Premix^a^	1.50
Nutrient level	
Crude protein	16.82
DE, MJ/kg	13.61
Lys, %	1.15
Met, %	0.56
Thr, %	0.54
Trp, %	0.16

a Provided per kilogram of mixed diet: vitamin A, 8,000 IU; vitamin D_3_, 350 IU/; vitamin E, 24 mg; vitamin B_1_, 2.6 mg; vitamin B2, 3.0 mg; vitamin B6, 1.8 mg; vitamin B12, 0.18 mg; vitamin K3 2.5 mg; folic acid 0.5 mg; biotin 0.18 mg; pantothenic acid 7 mg; nicotinic acid 7 kg; copper 10 mg; zn 70 mg; Fe 90 mg; I 0.3 mg; Mn 12 mg; Se 0.6 mg.

**Table 2.** Main components of Astragalus dregs (%)

Active ingredient	Astragalus dregs
polysaccharides	13.5
total flavoniods	0.27
saponin	0.15

### Determination index

#### Body weight, daily feed intake of fattening pigs in 0–28 days

The fattening pigs were weighed on the morning of 0th and 28th of the experiment. Daily feed intake was calculated based on the weight of the diet (08:00 AM and 08:00 PM daily).

#### Diarrhoea rate of fattening pigs

Observe faeces every morning and evening, record the number of diarrheal pigs in detail and score faeces for the scoring standard.

#### Apparent digestibility test

On the 24th to 28th day of the test, the faeces collection was started after 1 h of feeding every morning.

#### Blood indices

On the 28th day, after record the final body weight and ADG. Blood were collected from anterior vena cava with vacuum blood collection vessels, stayed at room temperature for 30 min, then centrifuged at 3500 rpm for 10 min, separated and stored at −20°C for blood indexes analysis. Biochemical parameters were measured using an automatic biochemical analyzer (selectra proxL, Netherlands). The antioxidant capacity was measured by superoxide dismutase (SOD), glutathione peroxidase (GSH-Px), total antioxidant capacity (T-AOC), and malondialdehyde (MDA) kits (Nanjing Jiancheng Biotechnology, Nanjing, China). The serum levels of immunoglobulin A (IgA), immunoglobulin G (IgG), immunoglobulin M (IgM), interleukin-2 (IL-2), interleukin-6 (IL-6) and interleukin-10 (IL-10), interleukin-22 (IL-22) were measured using ELISA kits (Yuanye Biotechnology, Shanghai, China).

### Determination method

#### Growth performance of fattening pigs

Calculated according to the following formula, average feed intake (ADFI) = whole feed intake (kg)/days/(d), average daily weight gain (ADG) = whole weight gain (kg)/days (d), feed to weight ratio (F/G) = cumulative feed consumption (kg)/(final weight-initial weight) kg, Diarrhoea rate (%) = [number of pigs with diarrhoea per stall/number of pigs per stall × days] × 100%.

#### Diarrhoea rate of fattening pigs

Faeces were observed every morning and evening, and the number of diarrhoea was recorded in detail. Evaluation was assessed using a 5-grade scoring assessment. The scoring criteria were as follows: 1 = compact, dry pellets in a small, firm mass; 2 = firm, shaped stool that retains firmness and softness; 3 = soft, shaped, and moist faeces that maintain form; 4 = tender, shapeless faeces that conform to the container’s shape; and 5 = fluid, watery faeces that can be poured.

#### Determination of the Astragalus polysaccharide content of Astragalus dregs

Weighing 30 g Astragalus dregs, after high-speed pulverised, Astragalus polysaccharides were extracted by rotary evaporator at 100 °C, solid-liquid ratio was 1: 16. The collection time was 2 h for 3 times evaporation, the product was added 95% ethanol until the final concentration is 70%. Stay at room temperature for 12 h and calculate polysaccharide yield after collecting sediment and dryed.

#### Apparent digestibility test

Digestibility was determined by hydrochloric acid insoluble ash method. Collect faeces by replicate unit, with 300 g of faeces per replicate, and immediately place them in refrigerator at −20 °C for storage.

#### Blood indices measurement

Biochemical parameters were detected by automatic biochemical analyzer (selectra proxL, Netherland), antioxidant capacity and immune factors were analysed by the instructions of the Elisa kits.

## Statistical analyses

An independent sample *t*-test method was used to compare the values between the control and experimental groups using SPSS 22.0 statistical software (SPSS Inc., Chicago, IL, USA). The results are shown as mean±S.E. The test level α = 0.05 and *P* < 0.05 was considered statistically significant. 0.05 *< P* *<* 0.1 was considered to have a statistical trend.

### Result

#### Growth Performance and Diarrhoea rate

As shown in Table 3, compared with the control group, the ADG, ADFI and F:G of pigs supplemented with Astragalus dregs were increased and diarrhoea rate tended to be lower (*P* > 0.05).

**Table 3.** Effects of Astragalus dregs on growth performance and diarrhoea rate in fattening pigs

Item	Initial Body Weight (kg)	Final Body Weight(kg)	ADG (kg/d)	ADFI (kg/d)	F:G	Diarrhoea rate %
Control	75.20 ± 2.63	85.70 ± 2.36	0.37 ± 0.09	2.48 ± 0.56	5.89 ± 0.89	0.88 ± 0.11
10% Astragalus dregs	75.30 ± 1.81	86.88 ± 2.18	0.416 ± 0.10	2.37 ± 0.42	5.69 ± 0.57	0.22 ± 0.05

#### The yield of Astragalus polysaccharides

The yield of Astragalus polysaccharides was 11.93%.

#### Apparent digestibility of feed nutrients in fattening pigs

As shown in Table 4, the digestibility of dry matter, crude protein and ether extract were significantly improved in fattening pigs (*P* *<* 0.05).

**Table 4.** The effect of Astragalus dregs on apparent digestibility of feed nutrients in fattening pigs

Item	control	10% Astragalus dregs
DM(%)	83.35 ± 3.95^a^	80.78 ± 4.15^b^
EE(%)	54.97 ± 3.72^a^	48.36 ± 3.89^b^
CP(%)	79.52 ± 4.36^a^	75.74 ± 3.28^b^
CF(%)	40.08 ± 2.72	41.83 ± 2.93
GE(%)	81.64 ± 5.42	81.86 ± 4.67

*Note:* ^a,b^Mean values within a row with different superscript letters are significantly different (*p* < 0.05).

#### Blood biochemical parameters

As shown in Table 5, compared with the control group, the globulin and total protein were increased in fattening pigs supplemented with Astragalus dregs (*P* < 0.05); the triglyceride and cholesterol were decreased in fattening pigs supplemented with Astragalus dregs (*P* < 0.05).

**Table 5.** The effect of Astragalus dregs on blood biochemical parameters in fattening pigs

Item	control	10% Astragalus dregs
Glucose(mmol/L)	4.83 ± 0.19^a^	4.75 ± 0.18^b^
Globulin(g/ L)	26.00 ± 2.21^b^	28.95 ± 1.11^a^
Cholesterol(mmol /L)	1.21 ± 0.06^a^	1.12 ± 0.04^b^
Triglyceride(mmol /L)	0.43 ± 0.02^a^	0.36 ± 0.03^b^
Total protein(g/L)	65.07 ± 1.91^a^	68.16 ± 1.11^b^

*Note:* ^a,b^Mean values within a row with different superscript letters are significantly different (*p* < 0.05).

#### Immunity

As shown in Table 6, compared with the control, the IgA, IgG and IgM contents were significantly increased by Astragalus dregs in fattening pigs (*P* < 0.05). The IL-2 and IL-6 content were significantly reduced (*P* < 0.05), and the IL-10 and IL-22 content were increased (*P* < 0.05) by Astragalus dregs in fattening pigs.

**Table 6.** Effects of Astragalus dregs on Immunity in fattening pigs

Item	control	10% Astragalus dregs
IgA(μg/mL)	15.44 ± 1.12^b^	20.77 ± 0.25^a^
IgG(mg/mL)	9.44 ± 0.47^b^	16.15 ± 0.98^a^
IgM(mg/mL)	7.25 ± 0.37^b^	16.25 ± 0.98^a^
IL-2(pg/mL)	35.67 ± 3.54^b^	23.77 ± 1.01^a^
IL-6(pg/mL)	112.43 ± 4.19^b^	97.51 ± 4.32^a^
IL-10(pg/mL)	93.33 ± 0.57^a^	112.33 ± 2.08^b^
IL-22(pg/mL)	122.33 ± 5.85^b^	134.00 ± 6.24^a^

*Note:* ^a,b^Mean values within a row with different superscript letters are significantly different (*p* < 0.05).

#### Antioxidant capacity

In Table 7, compared with the control group, the SOD, GSH-Px and T-AOC activities were significantly increased (*P* < 0.05) by Astragalus dregs, and the MDA content was significantly decreased (*P* < 0.05) by Astragalus dregs.

**Table 7.** Effects of Astragalus dregs on antioxidant capacity in fattening pigs

Item	control	10% Astragalus dregs
GSH-pX(U/mL)	128.71 ± 1.23^b^	150.24 ± 3.43^a^
SOD(U/mL)	44.87 ± 2.68^b^	58.37 ± 0.78^a^
T-AOC(U/mL)	12.75 ± 0.93^b^	16.55 ± 0.32^a^
MDA(μmol/mL)	1.56 ± 0.79^b^	1.22 ± 0.21^a^

*Note:* ^a,b^Mean values within a row with different superscript letters are significantly different (*p* < 0.05).

## Discussion

As the residue after the extraction of principal components, Astragalus dregs have emerged as a prominent topic of research in recent years. The development and utilisation of Astragalus dregs can enhance the overall utilisation rate of Astragalus and reduce the environmental pollution. Studies have indicated that Astragalus dregs possess the potential to bolster the resistance and immune response of livestock to diseases, consequently leading to a reduction in mortality rates^(9,19,20)^. Digestibility test results of our findings indicated that the supplemented Astragalus dregs to the basal diet can improve nutrient digestibility of fattening pigs (Table 4). The supplementation of 15–30% Astragalus dregs to the diets of both infant and adult goats has been demonstrated to elevate the levels of essential amino acids and α-linolenic acid while reducing muscle water loss. In the present study, it showed that Astragalus dregs can improve the growth performance of fattening pigs. This finding is consistent with the work of S. L. et al. (2009), who reported that Astragalus powder, as an additive, enhances both growth performance and immune function in pigs^(21)^. Our analysis revealed that the Astragalus dregs used in this study contain 11.93% Astragalus polysaccharides. Astragalus polysaccharides are known to facilitate the digestibility and absorption of nutrients^(22)^, which led to the observed improvement in the growth performance of the fattening pigs in our study.

Serum biochemical indices are integral parameters for assessing the nutritional and physiological status of animals. These indices reflect animal’s metabolic activities, organ function, and the efficacy of dietary interventions. Quan et al. (2004) reported serum total protein content was elevated in bamboo rats fed a diet supplemented with 600 mg/kg Astragalus polysaccharide^(23)^. This observation was consistent with the total protein content increase in the Astragalus dregs group in our study. The increase in total protein content may be attributed to the robust protein metabolism within the body. Liqiang Ji et al. (2017) found that the serum content of CHO and TG were reduced by Astragalus polysaccharide in the ZDF rats of type 2 diabetic, indicating that Astragalus polysaccharide can regulate the lipid metabolism process of animals, which is consistent with the results of our present study^(24)^. In our present study, it may be the component of Astragalus polysaccharide in Astragalus dregs that decrease TG and CHO content in blood of fattening pigs.

The antioxidant enzyme activity was increased and the content of MDA in serum was significantly decreased in our findings. Studies showed that the Astragalus polysaccharide and flavonoids in Astragalus dreg could increase the activities of SOD, GSH-px and decrease the content of MDA in the organism. The results showed that the antioxidant active ingredients in the residue of Astragalus could significantly improve the antioxidant capacity of the body.

Xiao Shunhan et al. (2009) reported that Astragalus polysaccharide can improve the level of cytokines in mouse serum; high concentrations of Astragalus extract can inhibit the induction of cytokine IL-2^(25)^. In the present study, Astragalus dregs reduced the levels of IL-2 and IL-6, which may be due to the bidirectional effect of Astragalus polysaccharide on immune regulation. Studies showed that polysaccharides in Astragalus can inhibit the expression of NF-KB-induced inflammatory factors by regulating TLR4 receptor^(26)^. IL-6 stimulates IL-1β secretion and plays a major role in the acute phase of inflammation. Anti-inflammatory cytokines include IL-10 and IL-22, etc.,. IL-10 is mainly secreted by CD4^+^Th2 cells and B lymphocytes, and it inhibits macrophages from expressing pro-inflammatory cytokines to exert anti-inflammatory functions at multiple levels. IL-22 is a member of the IL-10 family of cytokines. IL-10 and IL-22 can inhibit inflammation, and protect gastrointestinal tract health.

Immunoglobulin G (IgG) is the predominant antibody component in serum, constituting approximately 75% of the total immunoglobulin content. IgG is capable of activating the complement system, facilitating the phagocytic activity of engulfing cells, and participating in antibody-dependent cell-mediated cytotoxicity reactions. IgM is the earliest antibody produced in the body. IgA mainly plays a role in mucosal immunity. Enhanced intestinal immune function exert anti-virus and anti-bacteria effect with diarrhoea rate reduction. Xiao et al. (2010) found that the content of immunoglobulin was increased in dogs injected with Astragalus polysaccharide^(27)^. Astragalus polysaccharides can serve as immunomodulator, enhancing the immune function of animals.

As feed additive, Astragalus dregs is a potentially immunomodulator with many natural component, low production cost and wide application. Application of Astragalus dregs provide a new method to healthy growth and immune-enhancing in pig for pig farmers. The economic and social benefits of implementing Astragalus as a health-promoting feed additive are sustainable in the livestock industry.

## Conclusion

Astragalus dregs at a 10% level in the diet of fattening pigs has been demonstrated to enhance immune function and antioxidant capacity, potentially improving growth performance. As a green and health-promoting feed additive, Astragalus dregs broadens the research scope of functional feed. The optimum supplementation ratio of Astragalus dregs to the basal diet need further research and discussion.

## Supporting information

Guo et al. supplementary material Guo et al. supplementary material

## Data Availability

Supporting data for this study will be shared upon request by the corresponding author.

## Supplementary material

The supplementary material for this article can be found at https://doi.org/10.1017/jns.2024.95
