# Supplementary material for: Supplemented with Astragalus dregs improves growth performance, immunity, and antioxidant capacity in fattening pigs
Source: J Nutr Sci. 2025 Jan 17;14:e5. doi: 10.1017/jns.2024.95 (PMC11811845; doi:10.1017/jns.2024.95)
Supplement: Guo et al. supplementary material [file S2048679024000958sup001.docx]

Supplementary Table 1 Dietary composition, and nutrient level of diet

| Item |  | control | 10% Astragalus dregs |
| --- | --- | --- | --- |
| Ingredient, %  Corn |  | 65.00 | 65.00 |
| Soybean meal, fermented |  | 20.00 | 20.00 |
| Bran |  | 8.00 | 8.00 |
| Fish meal |  | 2.00 | 2.00 |
| Calcium hydrogen phosphate |  | 0.60 | 0.60 |
| Sodium chloride |  | 0.70 | 0.70 |
| Limestone |  | 0.70 | 0.70 |
| Soybean oil |  | 0.50 | 0.50 |
| Premix^1^ |  | 1.5 | 1.5 |
| Nutrient level  Crude protein |  | 16.82 | 16.86 |
| DE, MJ/kg |  | 13.61 | 13.61 |
| Lys, % |  | 1.15 | 1.15 |
| Met, % |  | 0.56 | 0.56 |
| Thr, % |  | 0.54 | 0.54 |
| Trp, % |  | 0.16 | 0.16 |

^1^Provided per kilogram of mixed diet: vitamin A, 8,000 IU; vitamin D_3_, 350 IU/; vitamin E, 24 mg; vitamin B_1_, 2.6 mg; vitamin B2, 3.0 mg; vitamin B6, 1.8 mg; vitamin B12, 0.18 mg; vitamin K3 2.5 mg;folic acid 0.5mg; biotin 0.18mg; pantothenic acid 7mg; nicotinic acid 7kg;copper 10mg;zn 70mg;Fe 90mg;I 0.3mg;Mn 12mg;Se 0.6mg.
